# Effects of Rocky Desertification Stress on Oat (*Avena sativa* L.) Seed Germination and Seedling Growth in the Karst Areas of Southwest China

**DOI:** 10.3390/plants13223260

**Published:** 2024-11-20

**Authors:** Haiyan Huang, Yuting Yang, Junqin Li, Yang Gao, Xiangtao Wang, Rui Wang, Zijun Zhou, Puchang Wang, Lili Zhao

**Affiliations:** 1 School of Life Sciences, Guizhou Normal University, Guiyang 550025, China; 15186985817@163.com (H.H.); yutingyang0509@163.com (Y.Y.); lijq489@nenu.edu.cn (J.L.); wxt_11@163.com (X.W.); 13118563086@163.com (R.W.); 15085781094@163.com (Z.Z.); 2 School of Karst Science, Guizhou Normal University, Guiyang 550025, China; gyphoebe945@126.com; 3 College of Animal Science, Guizhou University, Guiyang 550025, China; zhaolili_0508@163.com

**Keywords:** oats, seed germination, seedling growth, drought, pH, CaCl_2_, rocky desertification adversity

## Abstract

Oat is an important crop widely distributed in temperate zones and is also commonly planted in the karst areas of southwest China. However, due to severe rocky desertification, the complex soil in this area is characterized by high calcium content, alkaline conditions, and drought, which significantly negatively impact the growth of oat seedlings. To study the adaptability of oats to rocky desertification stress at the seedling stage, we investigated the effects of CaCl_2_ (0–150 mM), the pH (3–9), and drought stress (PEG-6000 solution at 0 to −0.79 MPa) on seed germination and seedling growth. The results showed that (1) calcium stress had dual effects on seed germination within the range of 5–150 mM CaCl_2_. Low concentrations of CaCl_2_ (5 mM) promoted the germination potential, germination rate, germination index, and vigor index of oats, as well as the growth and biomass accumulation of radicles in oat seedlings; however, high concentrations of CaCl_2_ inhibited these germination parameters. (2) Under drought stress, moderate concentrations of a PEG-6000 solution significantly improved the germination potential and germination rate of oat seeds, but the germination index and vigor index decreased with an increasing PEG-6000 concentration. When the PEG-6000 concentration corresponded to −0.06 MPa, the root growth and fresh weight accumulation of oat seedlings were significantly promoted; however, as the concentration increased to −0.53 MPa and –0.79 MPa, seed germination and seedling growth were significantly inhibited. (3) pH treatments had no significant effect on oat seed germination, but all growth indexes of oats showed a downward trend under alkaline conditions. These results suggest that suitable conditions for oat planting in karst rocky desertification areas are 5 mM CaCl_2_, pH levels of 5–8, and drought stress between 0 and −0.32 MPa. This study provides a theoretical basis for oat introduction, cultivation, and stress-resistant breeding in this area.

## 1. Introduction

The southwest China karst region, the center of karst landforms nationally, covers an area exceeding 500,000 square kilometers and encompasses a wide range of soil types, including yellow loam, limestone soils, and yellow paddy soils [1]. Similarly to other fragile ecosystems, this area is suffering from severe rocky desertification caused by both natural and anthropogenic factors [2]. Rocky desertification further deteriorates soil characteristics, leading to increased calcium content, alkalinity, and drought conditions, which become major stressors affecting seed germination [3]. In karst areas, soils are extremely rich in calcium ions, with concentrations more than three times those in acidic soils [4]. Specifically, the calcium content in loamy sandy soils, silts, and clays is 300, 650, and 700 mg/kg, respectively [5], while in typical karst areas of Guizhou, the soil calcium content can be as high as 1500 mg/kg [6]. Additionally, karst areas suffer from frequent droughts due to a poor soil water-holding capacity, leading to a series of physiological and biochemical reactions in plants caused by water deficits, such as protoplasmic dehydration and metabolic disorders that affect normal plant growth [7]. With the intensification of rocky desertification, the depletion of CO_2_ and the dissolution of calcium carbonate in the soil lead to an increase in the pH [8]. The pH, as an important environmental factor, has a significant effect on the growth and development of plants. In summary, high calcium, drought, and the soil pH in karst areas are the main stress factors limiting plant growth and crop yields. Therefore, the study of plant adaptation to calcium, drought, and pH stresses in karst regions, as well as the breeding of crop and cash crop varieties that can tolerate these stresses, is essential to promote the development of sustainable agriculture in these regions.

In karst rocky desertification areas, plant growth and development are affected by multiple factors such as the salinity, water availability, and soil pH, especially during seed germination and seedling establishment [9,10]. Drought and saline–alkali stress often hinder plant physiological activities, affecting normal growth and reproduction [10,11]. Under drought conditions, plants initiate a series of physiological and biochemical reactions due to water scarcity, leading to protoplasmic dehydration and metabolic disorders that affect plant growth [12,13]. For example, when the soybean encounters drought stress during seed formation or grain filling, the seedling vitality and germination rates are affected [14]. Studies have shown that seed germination and seedling axial dry weight decrease with increasing drought stress intensity [15,16]. Drought stress can also cause reactive oxygen species (ROS) damage, leading to stomatal closure, restricted leaf growth, and enhanced photorespiration [17], thus interfering with normal plant growth and development [18]. Plants can reduce water loss by closing stomata (reducing stomatal conductance, gs) and inhibiting photosynthesis (Pn) and transpiration (E) to cope with a soil water deficiency. Osmoregulation and the accumulation of secondary metabolites in plants (e.g., flavonoids and polyphenols as well as APX, SOD, CAT, and GR of the antioxidant enzyme system that responds to oxidative stress) help to mitigate the adverse effects of drought [19,20].

The calcium ion (Ca^2+^) is not only an essential mineral element for plants but also a key regulator of plant growth and development and an important component of plant cell walls. Calcium ions play a crucial role in plant development and can help plants resist damage from various stresses. However, calcium-rich soils may cause cells to take up more calcium than needed [21,22]. In karst areas, the soil calcium content is generally high [23], and it has been found that the soil calcium content increases with the degree of rocky desertification, indicating a significant effect of desertification on soil calcium levels. Wei (2018) selected mild, moderate, and severe rocky desertification sample areas in the karst region of southwest China and found that the soil exchangeable calcium content was 0.02 g/kg in mildly affected areas, while it was as high as 3.92 g/kg in severely affected areas [24]. Soils with high calcium content in rocky desertification areas have become one of the most important environmental factors affecting the physiological characteristics and distribution of local plants. Excessive calcium ions not only inhibit plant growth but also affect crop yields. For most plants, seed germination and the seedling stage are the most vulnerable and critical stages of growth, and their ability to adapt to stress determines their distribution and survival chances. In high-calcium karst environments, the specialization of plant stress tolerance is particularly important. Studies have shown that high concentrations of calcium ions can inhibit seed germination, interfere with plant photosynthesis, and reduce plant growth characteristics [25], which in turn inhibits overall plant growth. Moreover, an excess of intracellular calcium ions can lead to cell dysfunction and even cell death [26]. Therefore, maintaining the balance of intracellular calcium ions is crucial for normal plant growth. Especially for plants growing in high-calcium environments, possessing specific physiological mechanisms to prevent the over-absorption of calcium ions is essential [27]. Seed germination is also affected by the pH value, especially in calcareous and sodic soils [28]. A high pH may indirectly lead to deficiencies of essential elements such as phosphorus (P), iron (Fe), and zinc (Zn) and cause toxicity through the accumulation of bicarbonate (HCO^3−^) [29]. This is because excessive bicarbonate accumulation in an overly alkaline environment leads to the hydrolysis of CO₃^2−^ and HCO₃⁻ ions, which is toxic to seeds. Additionally, high concentrations of hydroxide ions (OH^−^) at elevated pH values directly inhibit plant root elongation [30].

Oat (*Avena sativa* L.) has been one of the most important food crops in recent years due to its high nutritional value and adaptability to various soil conditions. It is widely cultivated in temperate regions of the world, especially in high-altitude arid and cold areas in northwest, north, and southwest China [31]. In the karst areas of southwest China, oat has become an important crop for poverty alleviation because of its strong drought tolerance, cold tolerance, and salt and alkali tolerance. However, the rocky desertification process in this area leads to the enrichment of soil calcium salts and drought, which limits the stability of high oat yields. Studies have shown that the seed germination and early seedling growth (such as the root cap length, root cap fresh weight, and dry weight) of oat decrease with increasing drought and saline–alkali stress intensity [32,33]. In semi-arid and saline–alkali areas, oat is more tolerant than other crops and is considered a moderately salt-tolerant crop that performs better than other food crops such as wheat and rice [34,35].

In this study, we simulated the relationship between seed germination and stress size under drought, pH, and calcium stress in a karst rocky desertification area. Our main objectives are to evaluate the correlation between these stress factors and seed germination and seedling growth, determine whether these correlations depend on the variations in these stresses, and establish the range and indices of drought tolerance, calcium tolerance, and the pH concentration. This research aims to provide a theoretical basis for the early identification of drought-tolerant and calcium-tolerant plants and offer a reference for oat production and breeding for drought and salt tolerance. However, key questions remain unanswered: (1) How does oat cope with the multiple stresses of high-calcium, arid, and alkaline soils in rocky desertification areas? (2) Are there differences in the gene expression and physiological responses of oats under different stresses and their interactions that need to be further explored?

To address these questions, we hypothesize that strategies for oat adaptation to stress in rocky desertification areas include the regulation of the calcium ion concentration, the accumulation of osmoregulatory substances (e.g., proline, betaine), and the enhancement of antioxidant enzyme activities (e.g., superoxide dismutase, peroxidase) in response to calcium toxicity, drought, and pH changes. Furthermore, calcium, drought, and pH stresses may affect the cell structure and function by activating different signaling pathways and gene expression. These stresses might interact with each other; for example, drought could exacerbate calcium toxicity, and alkaline environments might reduce drought tolerance.

## 2. Materials and Methods

### 2.1. Plant Material

The oat (*Avena sativa* L.) variety ‘Madison’ was used in this study. Seeds were purchased from Beijing Zhengdao Seed Industry Co., Ltd. (Beijing, China).

### 2.2. Experimental Design

Three separate experiments were conducted to simulate the karst environmental stresses of drought, calcium-rich conditions, and varying pH levels on oat seed germination and seedling growth.

Simulated Karst Drought Stress: Polyethylene glycol (PEG-6000) solutions were prepared to create osmotic potentials of 0, −0.06, −0.17, −0.32, −0.53, and −0.79 MPa to simulate different levels of drought stress. PEG-6000 induces a water deficit by increasing the osmotic potential of the solution, thereby reducing the water availability to plant cells and mimicking drought conditions by inhibiting root water uptake.

Simulated Calcium-Rich Stress: Calcium chloride (CaCl_2_) solutions were used at concentrations of 0, 5 mM, 10 mM, 25 mM, 50 mM, 100 mM, and 150 mM to simulate the calcium-rich soil conditions typical of karst areas. The dissolution of CaCl_2_ increases the concentration of calcium ions (Ca^2+^) in the solution, thus replicating the high calcium content common in karstic soils.

Simulated pH Stress: The pH of the germination medium was adjusted using NaOH and HCl to create pH levels of 3, 4, 5, 6, 7, 8, and 9 to simulate the range of soil pH conditions found in karst environments. pH adjustments were monitored using pH test strips, and the desired levels were achieved by gradually adding NaOH or HCl solutions while continuously measuring until the target pH was reached.

The germination tests were conducted according to the standard method outlined in the Germination Test (GB/T 3543.4-1995) [36]. Madison oat seeds with a uniform size and fullness were selected for the experiments. The seeds were surface-sterilized with 1% sodium hypochlorite (NaClO) solution for 5 min, then rinsed twice each with tap water, distilled water, and deionized water. Excess surface water was removed using filter paper.

The treated seeds were placed in 9 cm diameter Petri dishes lined with double-layer filter paper. A volume of 5 mL of the respective PEG-6000 solution, pH-adjusted solution, or CaCl₂ solution was added to each dish using a pipette. The control group received the same amount of distilled water. Seeds were handled with sterilized tweezers (immersed in 75% ethanol), and 50 seeds were placed in each Petri dish. Each treatment was replicated four times (labeled as replicates A, B, C, and D). The dishes were covered and incubated at a constant temperature of 25 °C in an artificial climate chamber (Table 1).

### 2.3. Determination of Germination Indices

The experiment commenced on 4 September 2023. Germination was observed and recorded twice daily at 8:00 a.m. and 8:00 p.m. following the “International Rules for Seed Testing” (ISTA Rules) [37]. A seed was considered germinated when the radicle length was at least equal to half of the seed length. Evaporated water was replenished daily by weighing the Petri dishes and adding the required amount of water. Germination indices were recorded daily until the 14th day of incubation. The root length and shoot length were measured twice during the experiment. From each of the four replicates per treatment, 10 vigorous seedlings with similar growth were randomly selected. Surface moisture was blotted off with filter paper. They were initially heated at 75 °C for 30–40 min to halt metabolic activity, then dried at 105 °C for 8 h to a constant weight. The samples were weighed using an electronic balance with an accuracy of 0.0001 g to ensure data accuracy. Each treatment was repeated four times, and the average values were calculated. The total dry mass of seedlings was determined based on the measured data. Measured parameters and calculations:

Length of root (RL) and shoot length (SL): measured with a ruler.

Fresh weight (FW) and dry weight (DW): weighed with a 0.0001 g electronic balance.

Germination potential (GP) = (the number of seeds germinated in the first 4 days/the total number of tested seeds) × 100% [38].

Germination percentage (GR) = (the seed germination number the total seed number in 14 days/the total number of tested seeds) × 100%.

Germination index (GI) = Σ (Gt/Dt) vitality index [39].

Vitality index (VI) = GI × S.

TWC = (fresh weight-dry weight/fresh weight × 100%).

where Gt is the germination number on the t day; Dt is day t of germination; and S is the length of the germ.

### 2.4. Data Analysis

Raw data were organized using Microsoft Excel (version 2024). Analysis of variance (ANOVA) and Tukey’s multiple comparison tests were performed using IBM SPSS Statistics 22.0. A Pearson correlation analysis and heat mapping were conducted using the online platform https://www.chiplot.online (accessed on 15 October 2024). A one-way ANOVA was used to assess the differences in germination indices under different concentrations of PEG-6000, CaCl_2_, and pH treatments, with significance determined using the Tukey method at *p* < 0.05.

## 3. Results

### 3.1. Result Analysis

#### Effects of Different Stresses on Seed Germination of Oat

The germination potential (GP) of oat seeds initially increased and then decreased with an increasing CaCl₂ concentration (Table 2). At 150 mM CaCl_2_, the GP was significantly lower than the control (*p* < 0.05), while no significant differences were observed among the other treatments. When the calcium chloride concentration reached the highest treatment concentration, the germination percentage (70%) was significantly lower than that of the control (91%) and was significantly different (*p* < 0.05) from the other treatment groups (Table 2). The germination index was significantly lower (*p* < 0.05) than the control at 100 and 150 mM calcium chloride solution concentrations, while the other concentrations of calcium chloride treatments were not significantly different from the control. At 5 and 10 mM calcium chloride concentrations, the vigor index was not significantly different from the control, but there was a significant difference between the other treatment groups and the control (*p* < 0.05).

The GP of oat seeds showed a trend of increasing and then decreasing with changes in the pH (Table 3). The lowest GP was observed at pH 3, decreasing by 13.5% compared to the control. The GR exhibited an irregular trend with pH changes, reaching the highest value at pH 5; however, differences were not significant among treatments. The GI increased and then decreased with the pH, peaking at pH 7 (control), with significant differences observed at pH 3 and pH 4 compared to the control (*p* < 0.05). The VI varied irregularly with the pH and was significantly lower than the control at pH 3 and pH 9 (*p* < 0.05).

With an increasing PEG-6000 concentration (i.e., decreasing water potential), the GR of oat seeds first increased and then decreased (Table 4). The GR at −0.06, −0.17, and −0.32 MPa was higher than the control, but differences were not significant among treatments. At −0.79 MPa, the GR was significantly lower (28%) compared to the control (91%), representing a 69% reduction (*p* < 0.05). The GP was highest at −0.06 MPa (92%) and significantly decreased at −0.53 and −0.79 MPa compared to the control (*p* < 0.05), reaching a minimum of 16% at −0.79 MPa. The GI initially increased and then decreased with PEG-6000 treatment, with significant reductions observed at −0.53 and −0.79 MPa (*p* < 0.05). The lowest GI (3.23) was recorded at −0.79 MPa, representing a 79% decrease compared to the control. The VI decreased with an increasing PEG-6000 concentration, with significant differences among treatments (*p* < 0.05).

### 3.2. Effects of Different Stresses on Seedling Growth of Oat (Avena sativa L.)

#### 3.2.1. Effects of Calcium Stress on Oat (*Avena sativa* L.) Seedling Growth

**Figure 1:**
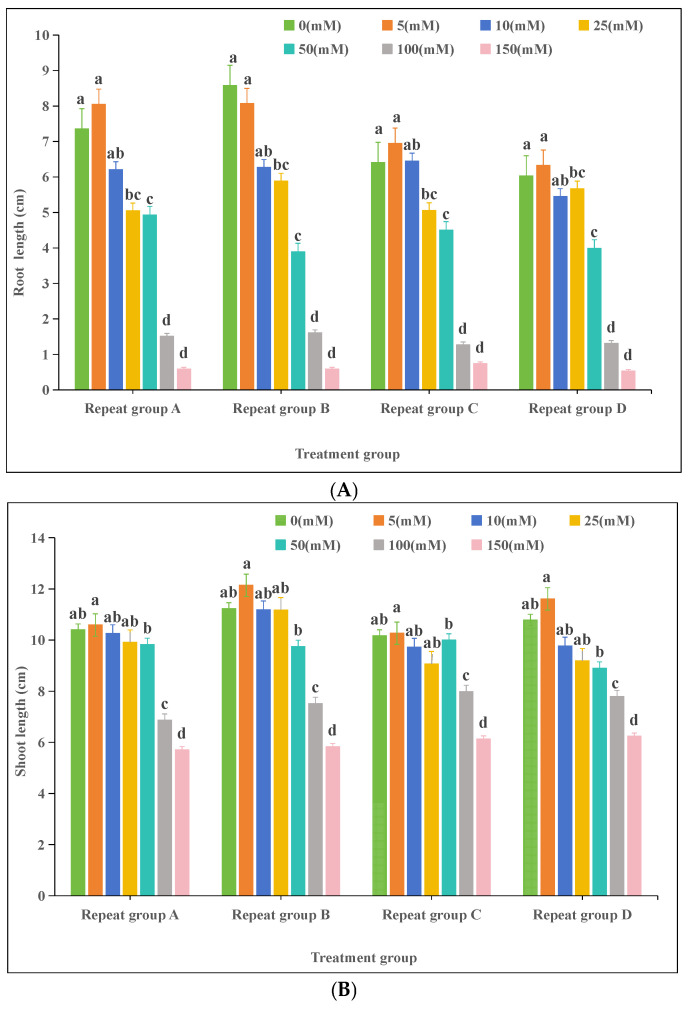
Effects of calcium stress on oat (*Avena sativa* L.) seedlings: (**A**) root length; (**B**) shoot length. Different lowercase letters in the same column indicate significant differences at *p* < 0.05; the same letter indicates no significant difference (*p* > 0.05).

In the experiment with varying CaCl₂ concentrations, the root and shoot lengths of oat seedlings exhibited an initial increase followed by a decrease (Figure 1). Root and shoot growth remained relatively normal at low CaCl_2_ concentrations. At 5 mM CaCl_2_, the average root length was 7.35 cm, 0.25 cm longer than the control, indicating the promotion of root growth. However, root growth was inhibited at higher concentrations. At 150 mM CaCl_2_, the root length was only 0.61 cm and the shoot length was 6.0 cm, representing decreases of 91% and 4.3% compared to the control. These results indicate that low concentrations of CaCl_2_ can promote seedling growth, while high concentrations significantly inhibit it (*p* < 0.05).

#### 3.2.2. Effects of pH on Oat (*Avena sativa* L.) Seedling Growth

**Figure 2:**
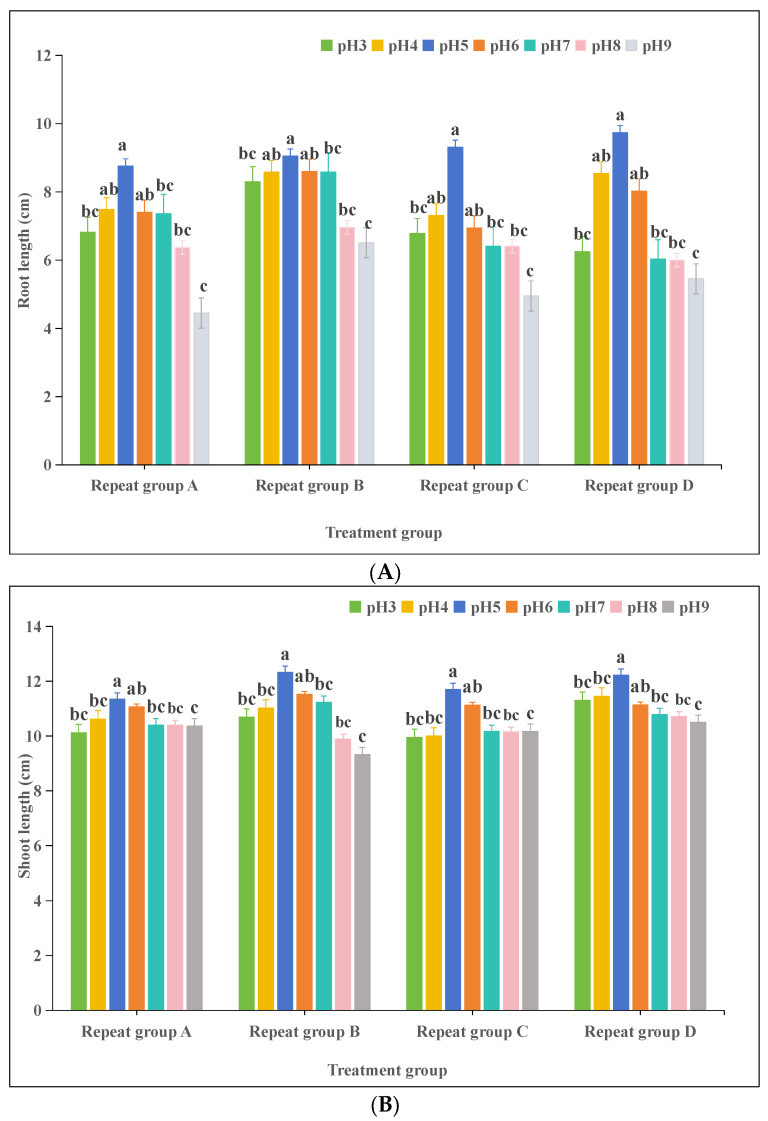
Effects of pH on oat (*Avena sativa* L.) seedlings: (**A**) root length; (**B**) shoot length. Different lowercase letters in the same column indicate significant differences at *p* < 0.05; the same letter indicates no significant difference (*p* > 0.05).

At pH 5, the mean root length of oat seedlings was significantly higher than the control (pH 7) (*p* < 0.05), reaching 9.22 cm. Other pH treatments did not significantly affect root length, except for a significant difference between pH 4 and pH 9. For the shoot length, a pH 5 treatment resulted in a significantly higher mean shoot length compared to the control (*p* < 0.05). Significant differences were also observed between pH 6 and pH 9 treatments, while other pH levels did not significantly affect shoot length, and no significant differences were found among those treatments (Figure 2).

#### 3.2.3. Effects of Drought Stress on Oat (*Avena sativa* L.) Seedling Growth

**Figure 3:**
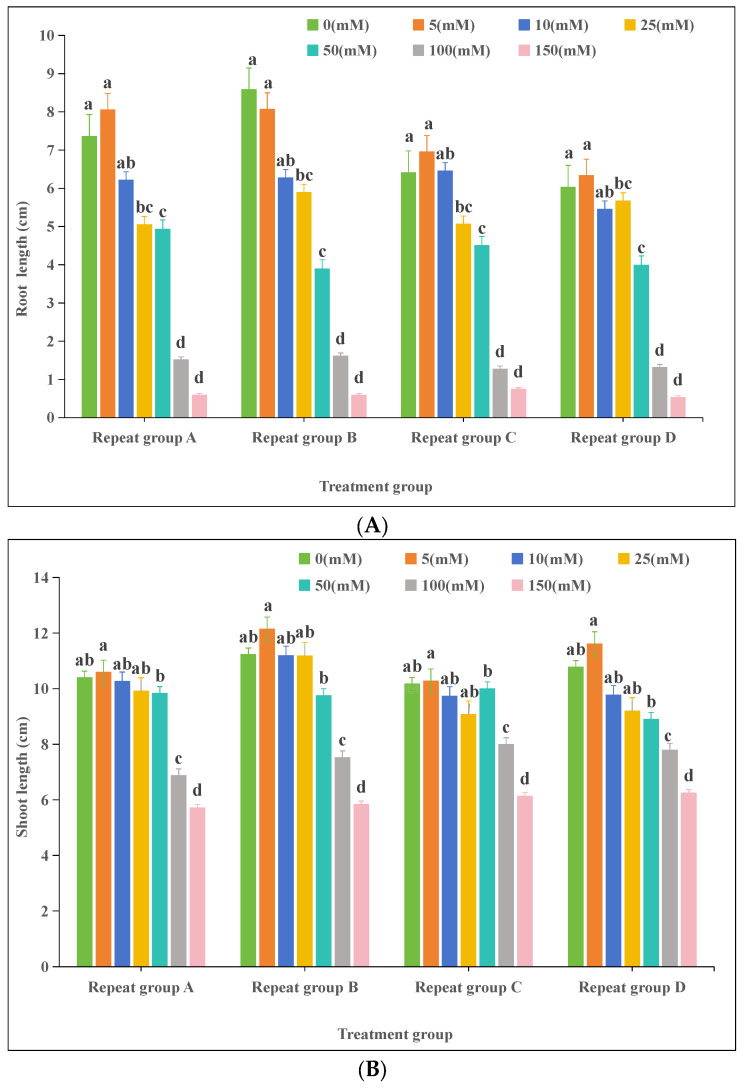
Effects of drought stress on oat (*Avena sativa* L.) seedlings: (**A**) root length; (**B**) shoot length. Different lowercase letters in the same column indicate significant differences at *p* < 0.05; the same letter indicates no significant difference (*p* > 0.05). Note: the unit of PEG-6000 (0 to −0.79) is MPa.

At a −0.06 MPa PEG-6000 treatment, the average root length reached 7.99 cm, which was higher than the control; however, the difference was not significant. With an increasing PEG-6000 concentration (decreasing water potential), the average root length decreased, and significant differences were observed between the control and treatments at −0.32, −0.53, and −0.79 MPa (*p* < 0.05). The shortest mean root length (2.15 cm) was observed at −0.79 MPa. For the shoot length, a gradual decrease was observed with an increasing PEG-6000 concentration (Figure 3B), and significant differences were found between all PEG-6000 treatments and the control (*p* < 0.05).

### 3.3. Effects of Different Stresses on Biomass and Tissue Water Content of Oat (Avena sativa L.)

**Figure 4:**
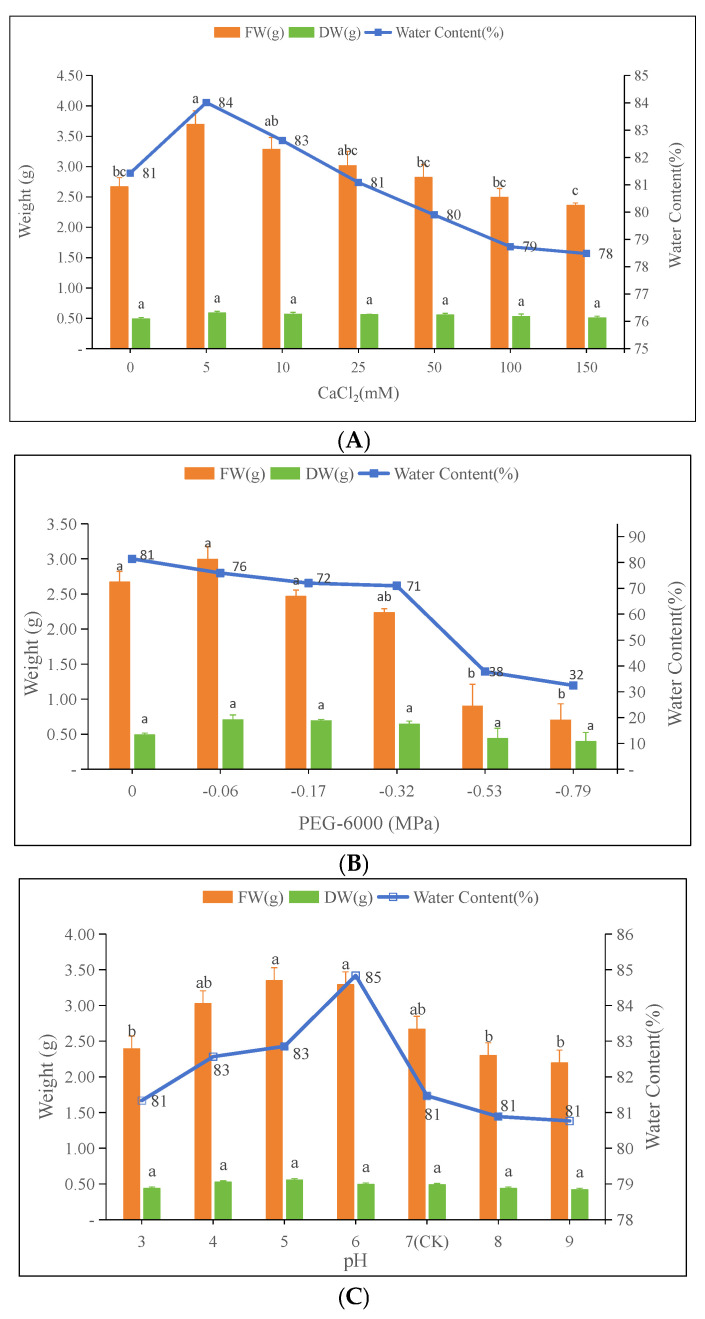
Effects of different stresses on oat (*Avena sativa* L.) biomass and water content: (**A**) calcium stress; (**B**) drought stress; (**C**) pH. FW: fresh weight; DW: dry weight. Data are presented as the mean ± standard error. Different letters indicate significant differences at *p* < 0.05.

As can be seen from Figure 4A, with an increasing CaCl_2_ concentration, the fresh weight initially increased and then decreased. The fresh weight at 5 mM CaCl_2_ was significantly higher than the control (*p* < 0.05), whereas no significant differences were observed in other treatments compared to the control. Among the treatments, significant differences in the fresh weight were observed between 10 mM and 50 mM CaCl_2_ concentrations (*p* < 0.05). For the dry weight, no significant differences were found among treatments or compared to the control across all CaCl_2_ concentrations.

Figure 4B shows that with an increasing PEG-6000 concentration, the fresh and dry weights initially increased and then decreased. At −0.06 MPa PEG, the fresh weight was 3.0 g and the dry weight was 0.7 g, 31% and 11% higher than the control, respectively. However, at −0.79 MPa PEG, the fresh weight, dry weight, and water content decreased significantly, with the water content dropping to 34.2%.

Figure 4C indicates that the effect of pH on fresh and dry weights initially increased and then decreased. At pH 5, fresh and dry weights peaked at 3.35 g and 0.56 g, respectively, with a moisture content of 85%.

### 3.4. Correlation of Growth Indices of Oat (Avena sativa L.) Seedlings Under Different Stresses

**Figure 5:**
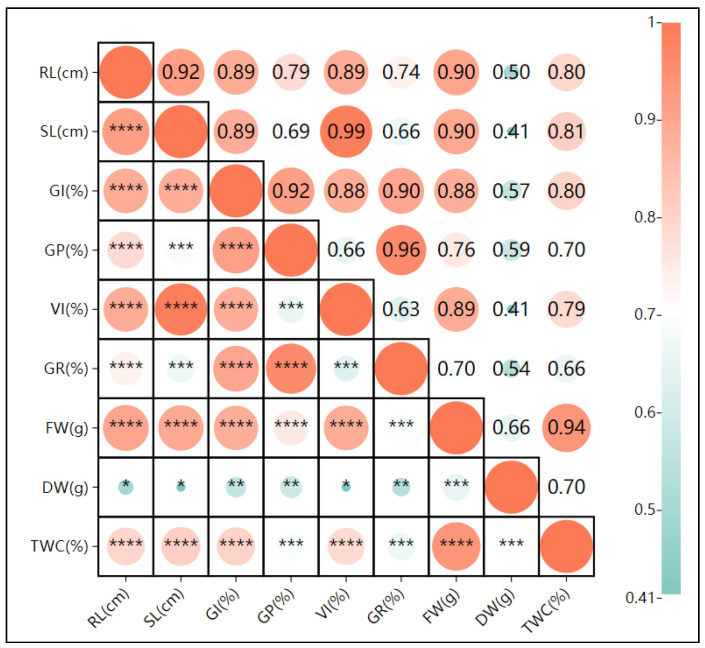
Pearson correlation analysis of the effects of calcium stress on oat (*Avena sativa* L.) seed germination. RL: root length; SL: shoot length; GI: germination index; GP: germination potential; VI: vigor index; GR: germination rate; FW: fresh weight; DW: dry weight; TWC: tissue water content. Note: **** indicates highly significant correlation at *p* ≤ 0.01; ***, ** indicates significant correlation at *p* ≤ 0.01, * indicates significant correlation at *p* ≤ 0.05.

Under drought stress (Figure 5), except for the significant correlation between the dry weight and root length, shoot length, and vigor index (*p* ≤ 0.05), all other growth indices showed highly significant correlations (*p* ≤ 0.01). For example, the germination potential (GP) and germination rate (GR) had a correlation coefficient of 0.96 ****, indicating that 96% of the information on seed germination is shared between GP and GR. This suggests these indices may synergistically enhance drought tolerance. The germination index was negatively correlated with the root length, shoot length, tissue water content, and fresh weight (r = −0.03, −0.05, −0.23, and −0.06).

**Figure 6:**
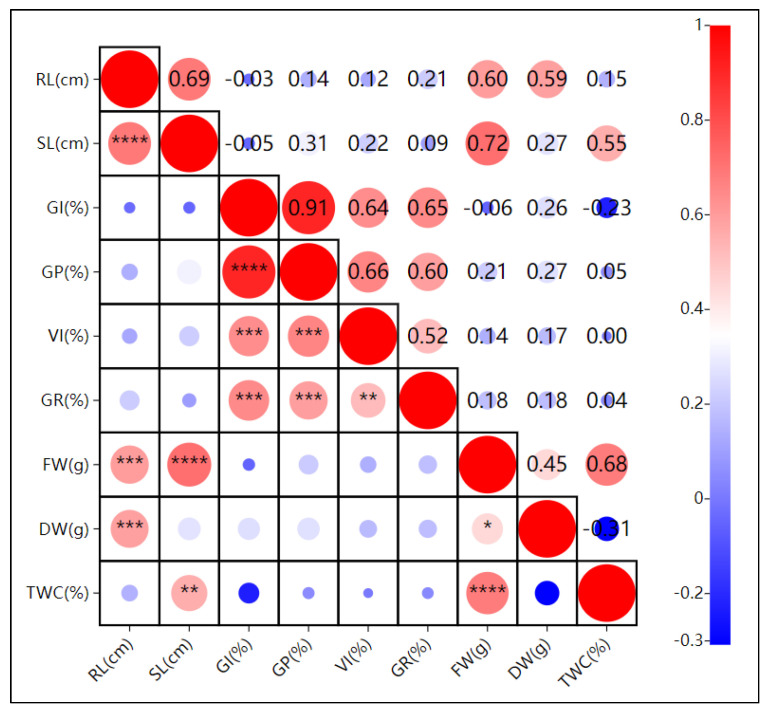
Pearson correlation analysis of the effects of pH stress on oat (*Avena sativa* L.) seed germination. RL: root length; SL: shoot length; GI: germination index; GP: germination potential; VI: vigor index; GR: germination rate; FW: fresh weight; DW: dry weight; TWC: tissue water content. Note: **** indicates highly significant correlation at *p* ≤ 0.01; ***, ** indicates significant correlation at *p* ≤ 0.01, * indicates significant correlation at *p* ≤ 0.05.

Under pH treatment (Figure 6), the germination index and germination potential were highly correlated (r = 0.91 ****), and other indices were also significantly correlated. The relationships between the root length and dry weight, shoot length and tissue water content, and fresh weight were highly significant (*p* ≤ 0.01), indicating that oat biomass can be stabilized or increased through root and shoot growth at specific pH levels. The root length and shoot length showed a significant positive correlation (r = 0.69, *p* < 0.01), suggesting consistent root and shoot growth responses to pH variations. Additionally, high positive correlations among the germination rate, germination index, vigor index, and germination potential indicate that oats maintain germination efficiency and vigor even under pH fluctuations.

**Figure 7:**
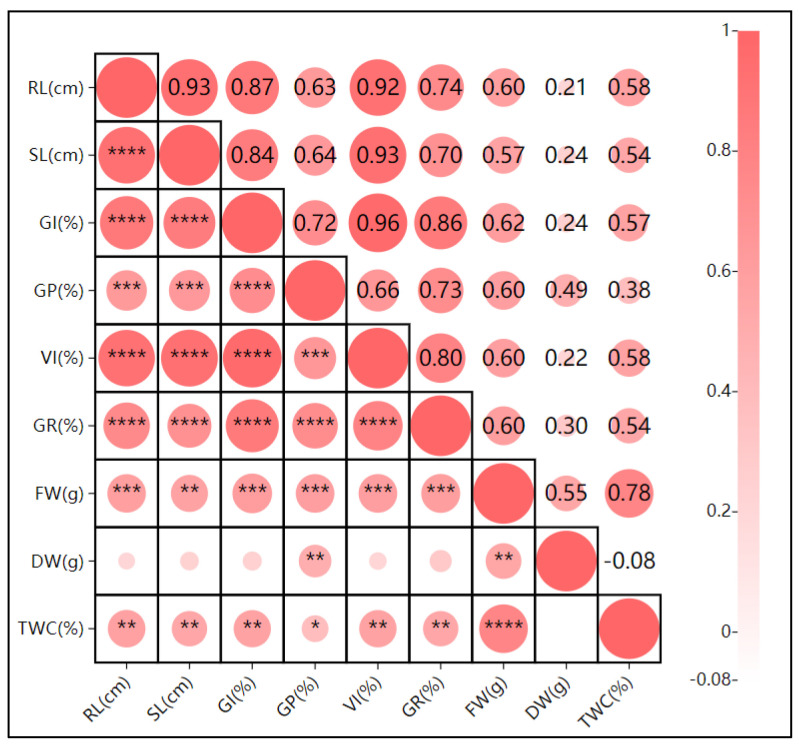
Pearson correlation analysis of the effects of drought stress on oat (*Avena sativa* L.) seed germination. RL: root length; SL: shoot length; GI: germination index; GP: germination potential; VI: vigor index; GR: germination rate; FW: fresh weight; DW: dry weight; TWC: tissue water content. Note: **** indicates highly significant correlation at *p* ≤ 0.01; ***, ** indicates significant correlation at *p* ≤ 0.01, * indicates significant correlation at *p* ≤ 0.05.

Under salt stress (Figure 7), the dry weight was significantly positively correlated with the root length, shoot length, vigor index, and germination rate (r = 0.21, 0.24, 0.24, 0.22, and 0.3), indicating that oats enhance survival under salt stress by promoting growth to accumulate dry matter. The negative correlation between the tissue water content and dry weight (r = −0.08) and the highly positive correlation between the tissue water content and fresh weight (r = 0.78 ***) suggest that oats adjust their water content to balance salt levels under salt stress. Other growth indicators also showed high positive correlations.

## 4. Discussion

### 4.1. Effect of CaCl_2_ Stress on Seed Germination of Oat (Avena sativa L.)

Karst landscapes, also known as limestone landscapes, are characterized by thin soil layers and high calcium content. In the karst areas of Guizhou Province, the soil calcium content is high and spatially heterogeneous, ranging from 1.045 g/kg to 28.437 g/kg, with a mean value of 11.38 g/kg [40]. In karst regions, excessive calcium ion stress significantly inhibits the growth of many plants and crop yields. It was shown that the germination of seeds was instead significantly increased when CaCl_2_ concentrations were 10, 20, and 30 mM, respectively [41]. In our experiment, the germination rate of oat seeds treated with a CaCl_2_ concentration of 5 mM was higher than that of the control group. However, as the CaCl_2_ concentration increased, the germination rate showed a decreasing trend (Table 2), indicating that oats have a certain degree of tolerance to calcium ions, but excessively high concentrations inhibit seed germination. Cai and Gao (2011) found that high concentrations of calcium ions (92.1 mM) in sewage sludge significantly inhibited radish seed germination and primary root growth [42]. Similarly, our results showed that high concentrations of calcium ions (100 mM and 150 mM) significantly inhibited the growth of oat roots and shoots, whereas low to medium concentrations (5–50 mM) did not have a significant effect on the root system and shoots of oats (Figure 1). This suggests that the soil calcium content should not be excessively high when cultivating oats to avoid negatively affecting the seedling growth and yield. Al-Whaibi et al. (2010) showed that the treatment of faba bean seedlings using different concentrations of a CaCl_2_ solution (0–100 mM) revealed that a concentration of 60 mM was most favorable for seedling growth [43]. When the treatment concentration was increased to 80 mM and 100 mM, the plant height, dry and fresh weights of above-ground parts, and dry weights of the roots of faba bean seedlings were significantly decreased compared to the 60 mM treatment group. In our study, the growth of oat seedlings was most favorable at a CaCl_2_ concentration of 5 mM. When the CaCl_2_ concentration was increased to 100 mM and 150 mM, oat seedlings showed a decreasing trend in the root length, shoot length, biomass, and tissue water content compared to the control and the 5 mM treatment groups (Figure 4), highlighting the importance of an appropriate CaCl_2_ concentration for promoting seedling growth. In summary, mild calcium ion stress had no significant effect on oat germination and might even promote it. However, heavy calcium ion stress inhibited seed germination, and the more severe the stress, the more significant the inhibition. This may be since the low concentration of calcium ion stress improved the osmotic regulation of the cell membrane, and the trace calcium ion stimulation promoted seed germination [44]. On the contrary, a high concentration of calcium ions produces osmotic stress and reduces the extracellular water potential, which affects the water uptake and swelling of seeds and prevents them from breaking through the seed coat, thus inhibiting germination [45].

### 4.2. Effect of Drought Stress on Oat (Avena sativa L.) Seed Germination

Karst areas face severe erosion and rocky desertification problems, and plants often suffer from short-term droughts even during the rainy season [46]. The shallow soils, porous limestone, low water-holding capacity, and strong rock permeability of the region cause frequent and intense alternating wet and dry stress [47]. The karst soil moisture content fluctuates between dry and rainy seasons, with an average range of 21.67–28.79%, and decreases with increasing rocky desertification. Wang et al. (2010) reported that the soil moisture content at different rocky desertification levels was as follows: light (30.67%), medium (28.52%), and potential (36.23%) [48]. Chen et al. (2008) determined the soil moisture characteristics of Guzhou Village, Huanjiang County, Guangxi, through centrifugation and concluded that the field moisture capacity was 0.03 MPa [49]. Drought stress became a key factor affecting plant survival in this area [50]. Studies have shown that moderate drought stress can stimulate the germination ability of certain plant seeds. For example, Tang et al. (2019) found that a 10% PEG-6000 solution (−0.32 MPa) treatment promoted the germination of Cannabis sativa L. seeds more than the control and 20% PEG-6000 solution (−0.53 MPa) treatments by simulating drought conditions [51]. Similarly, mild drought stress induced by 5% and 10% PEG-6000 solutions (corresponding to −0.028 MPa and −0.06 MPa, respectively) significantly increased the germination rate, germination index, and vigor index of Hibiscus sabdariffa seeds, surpassing the control and accelerating the germination process. However, the inhibition of germination became more pronounced with increasing PEG-6000 concentrations, and the higher the concentration, the more significant the decrease in germination parameters [52]. Our results align with these findings. We observed that PEG-6000 concentrations of −0.06, −0.17, and −0.32 MPa all contributed to the enhancement of oat seed germination (Table 4). A study by Guo et al. (2024) found that in a 15% PEG-6000 solution (−0.099 MPa), the first germination time of lily seeds was delayed, and both the germination index and the vigor index as well as the germination rate were decreased, whereas the germination index was enhanced under a 5% PEG-6000 solution (−0.028 MPa) treatment [53]. In our study, the −0.06, −0.17, and −0.32 MPa PEG-6000 solution concentrations increased the germination potential and germination percentage of oat seeds but reduced the germination and vigor indices, except for the −0.06 MPa treatment. Overall, the simulated drought level of a 5% PEG-6000 solution (−0.06 MPa) significantly promoted seed germination and optimized relevant germination parameters. In contrast, 10% (−0.17 MPa) and 15% (−0.099 MPa) PEG-6000 solution concentrations increased the germination percentage but delayed the time to first germination and decreased germination and vigor indices. It has been shown that drought stress causes a decrease in the plant height, root length, leaf area, and biomass of mountain plant seedlings [54]. In our experiment, the average root length of oats was higher than that of the control when treated with a PEG-6000 solution concentration of −0.06 MPa. However, as the PEG-6000 concentration increased, the root length gradually shortened, and the shoot length also decreased with increasing drought stress. These results are consistent with the aforementioned studies.

### 4.3. Effect of pH on Seed Germination of Oat (Avena sativa L.)

Soil properties in karst areas exhibit calcium-rich, alkaline soils with a high cation exchange capacity and base saturation [55]. It was found that the pH of soils with different degrees of rocky desertification ranged from 7.28 to 7.83, which is slightly alkaline with small variations. In contrast, the soils of non-stony desertification and intensely stony desertification environments were acidic, with pH values below 7 [56]. Plants require a suitable pH environment during germination and growth [57], and the range of pH adaptation varies among plant species. For example, the suitable pH for the seed germination of plants such as cactus grass, balsam, and wheat is around 7. Studies have shown that the pH has an important effect on seed germination and seedling growth [58]. In the case of goatgrass (*Leymus chinensis*), for example, studies have shown that seed germination and seedling growth can only proceed normally in the pH range of 7.49 to 9.14, and that too high or too low a pH is not conducive to plant survival [59]. Some studies have altered the pH in plant seed germination environments by simulating acid rain and found that small rye (*X Triticosecal*) and alfalfa (*Medicago sativa*) seeds germinate better in neutral to acidic environments [60]. In this study, we evaluated the effects of different pH concentration treatments on oat seed germination and seedlings. The results showed that the germination of oat seeds reached 93% at pH 5 and seedling growth was enhanced (Table 3 and Figure 2), indicating that the germination and seedling growth of oat seeds could proceed normally under this treatment condition. At pH 7, the germination index of oat seeds was high, indicating high seed vigor, but seed death occurred during the germination process, which is similar to the germination of many terrestrial plant seeds in similar environments [61]. In addition, it has been found that a low pH leads to the reduced seed germination of Arabidopsis thaliana, delayed germination, and the inhibited growth of seedling primary roots [62]. In our experiment, the growth of oat seedlings at pH 3 was significantly lower than that at pH 5. The post-incubation of germinated oat seedlings showed that too acidic or too alkaline pH treatments negatively affected root and shoot growth, but the differences were not significant compared to the control. Overall, different pH treatments had less effect on the growth of oat seedlings, and the differences between the pH treatments and the control were not significant, except that the average root and shoot lengths were significantly higher at pH 5. In summary, the results of this study showed that different pH treatments had no adverse effects on oat seed germination and seedling growth. In particular, the condition of pH 5 had the best effect on oat seed germination and promoted good seedling growth, suggesting it can be considered the most suitable pH level. The pH has a significant effect on nutrient uptake because different pH environments affect the solubility and uptake efficiency of nutrients by the plant root system. In acidic environments, certain metal ions (e.g., iron, zinc) are more soluble and easily absorbed by plants, while in alkaline environments, the solubility of these ions is reduced, which may lead to deficiency symptoms. Additionally, the pH affects microbial activity, which in turn alters the form and availability of nutrients in the soil, thus affecting nutrient uptake by plants [63].

With the aggravation of rocky desertification in karst areas, issues such as calcium-rich, alkaline soils and drought will become more prominent, imposing higher adaptability requirements on plant growth. Future research should focus on how plants adapt to these complex stresses at different life cycle stages to identify stress-tolerant species suitable for rocky desertification areas and to provide a theoretical basis for promoting the application of oats in rocky desertification areas, the development of agricultural economy, and the breeding of more resistant oat varieties.

## 5. Conclusions

This study examined the effects of rocky desertification stress on oat seed germination and seedling growth in karst areas by simulating soil conditions with different calcium concentrations (CaCl_2_: 0–150 mM), pH levels (3–9), and drought degrees (PEG-6000: 0 to −0.79 MPa). Oat seed germination decreased with an increasing CaCl_2_ concentration, with the 5 mM CaCl_2_ treatment being the most favorable for germination and seedling growth. At 150 mM CaCl_2_, germination was significantly inhibited (*p* < 0.05) and seedling growth was adversely affected. Low to medium concentrations of PEG-6000 slightly promoted germination, whereas high concentrations (−0.53 and −0.79 MPa) significantly inhibited both germination and growth (*p* < 0.05). Oat seeds exhibited a broad pH adaptability, with pH 5 being optimal for germination without negatively impacting seedling growth. The results of this study provide suitable conditions for oat cultivation in karst rocky desertification areas—for example, a calcium salt concentration of 5 mM CaCl_2_, pH levels between 5 and 8, and water stress ranging from 0 to −0.32 MPa. These findings also provide an important theoretical basis for the introduction and cultivation of oats in this area and for research on stress-resistant breeding.

## Figures and Tables

**Table 1:** Experimental treatment scheme.

Different Stresses and Concentrations			
CaCl_2_ (mM)	pH	PEG-6000 (MPa)	Control
5	3	5	Deionized water
10	4	10	
25	5	15	
50	6	20	
100	8	25	
150	9	-	

Note: the control treatment used distilled water for seed germination.

**Table 2:** Germination indices of oat (*Avena sativa* L.) seeds under calcium stress.

	CaCl_2_ Concentration (mM)	GP (%)	GR (%)	GI (%)	VI (%)
CaCl_2_	0	76 ± 0.03 a	91 ± 4.43 a	23.28 ± 1.67 a	247.14 ± 15.05 a
	5	82 ± 0.04 a	91 ± 1.29 a	23.41 ± 0.91 a	256.01 ± 16.53 a
	10	77 ± 0.04 a	88.5 ± 1.71 a	20.85 ± 0.38 a	215 ± 10.45 ab
	25	76 ± 0.02 a	87.5 ± 3.10 a	19.16 ± 1.21 a	187.81 ± 11.03 b
	50	75 ± 0.03 a	84 ± 2.58 ab	18.86 ± 0.90 a	183.86 ± 6.86 b
	100	69 ± 0.08 ab	79 ± 4.65 ab	13.7 ± 0.78 b	98.59 ± 6.21 c
	150	54 ± 0.04 b	70 ± 3.46 c	10.44 ± 0.56 b	65.40 ± 4.08 c

The data in the table are the mean ± standard deviation, *n* = 4. Different lowercase letters in the same column indicate significant differences at *p* < 0.05; the same letter indicates no significant difference (*p* > 0.05). GP: germination potential; GR: germination rate; GI: germination index; VI: vigor index.

**Table 3:** Germination indices of oat (*Avena sativa* L.) seeds under different pH conditions.

	pH Concentration	GP (%)	GR (%)	GI (%)	VI (%)
pH	7	76 ± 0.03 ab	91 ± 0.04 a	23.28 ± 1.67 a	247.14 ± 15.05 a
	3	64 ± 0.03 b	82 ± 0.02 a	16.55 ± 0.87 b	178.79 ± 4.24 b
	4	68 ± 0.02 ab	89 ± 0.03 a	18.11 ± 0.49 b	196.28 ± 7.3 ab
	5	76 ± 0.02 ab	93 ± 0.01 a	19.39 ± 1.14 ab	219.98 ± 10.51 ab
	6	80 ± 0.03 a	91 ± 0.01 a	19.63 ± 0.91 ab	219.68 ± 12.94 ab
	8	77 ± 0.07 ab	92 ± 0.02 a	20.11 ± 1.42 ab	213.46 ± 17.61 ab
	9	71 ± 0.03 ab	88 ± 0.04 a	18.68 ± 0.92 ab	191.45 ± 8.69 b

The data in the table are the mean ± standard deviation, *n* = 4. Different lowercase letters in the same column indicate significant differences at *p* < 0.05; the same letter indicates no significant difference (*p* > 0.05). GP: germination potential; GR: germination rate; GI: germination index; VI: vigor index. The control pH is 7.

**Table 4:** Germination indices of oat (*Avena sativa* L.) seeds under drought stress.

	PEG-6000 Concentration (MPa)	GP (%)	GR (%)	GI (%)	VI (%)
PEG-6000	0	76 ± 0.03 ab	91 ± 4.43 a	23.28 ± 1.71 a	247.14 ± 15.90 a
	−0.06	92 ± 0.01 a	98 ± 0 a	23.85 ± 0.79 a	206.61 ± 14.59 ab
	−0.17	89 ± 0.03 a	96 ± 1.50 a	23.13 ± 0.13 a	187.16 ± 2.59 b
	−0.32	88 ± 0.01 a	95 ± 1.29 a	20.44 ± 0.32 a	107.91 ± 6.78 c
	−0.53	70 ± 0.04 b	86 ± 1.41 a	13.15 ± 0.64 b	15.84 ± 5.01 d
	−0.79	16 ± 0.08 c	28 ± 11.17 b	3.23 ± 1.29 c	0.18 ± 0.18 d

The data in the table are the mean ± standard deviation, *n* = 4. Different lowercase letters in the same column indicate significant differences at *p* < 0.05; the same letter indicates no significant difference (*p* > 0.05). GP: germination potential; GR: germination rate; GI: germination index; VI: vigor index.

## Data Availability

The original contributions presented in the study are included in the article; further inquiries can be directed to the corresponding authors.
